# Evaluation of hepatitis C virus intrafamilial transmission among families with one index case, a pilot study from Fars province, Iran

**Published:** 2016

**Authors:** Kamran B. Lankarani, Maryam Ardebili, Masood Sepehrimanesh, Maryam Nejabat, Mohammad Amir Hemmati Rad, Seyed Younes Hosseini

**Affiliations:** 1 *Health Policy Research Center, Shiraz University of Medical Sciences, Shiraz, Iran*; 2 *Gastroenterohepatology Research Center, Shiraz University of Medical Sciences, Shiraz, Iran*; 3 *National Research Institute for Science Policy, Tehran, Iran *; 4 *Bacteriology and Viorology Department, Shiraz University of Medical Sciences, Shiraz, Iran*

**Keywords:** Hepatitis C virus, Intrafamilial transmission, Risk factors, Socioeconomic state

## Abstract

**Aim:**

Our aim was to survey the rate and risk factors for Hepatitis C virus interfamilial transmission among families with one index case.

**Background:**

The role of intrafamilial transmission in Hepatitis C virus epidemiology is still debated.

**Patients and methods:**

A cross-sectional study was conducted on 34 families (236 members) of HCV infected patients from Fars province, spring to summer 2013. All subjects were first evaluated for the risk factors of exposure and then their serum was checked for the presence of HCV antibody and the genome, using ELISA and PCR. The genotype of all PCR positive cases was also determined by a commercial assay. Two independent sample t test and Chi-Square test were used to compare groups together.

**Results:**

In 18 out of 34 families, HCV antibody was detected (52.9%) in new members. Among them, HCV transmission in 11 families (32%) was also confirmed by PCR. Having a history of intravenous drug abuse (P=0.006) and incarceration (P=0.01) showed to be important risk factors for interfamilial transmission. Hence, blade/needle sharing (P=0.016) just following molecular assay and sex (P=036) only in the serologic analysis were also determined as significant risk factors. Furthermore, based on serologic results, medium socioeconomic state was further associated with this manner of transmission (P=0.019 and P=0.328). Interestingly, among relatives, 13 cases were brothers while just 5 cases were couples. The genotypes 3a and 1a were more prevalent among the population.

**Conclusion:**

In conclusion, our finding highlighted a noticeable role of interfamilial transmission for HCV spread and supports the significant role of close relatives, especially brother relationship in this spread. Hence, the socioeconomic state was associated with the transmission rate of virus in the family.

## Introduction

 Chronic infection with hepatitis C virus (HCV) remains a global health problem, which affecting about 3% of the world population. It is considered among the chief causes of liver cirrhosis, hepatocellular carcinoma and liver transplantation worldwide (1). The epidemiologic pattern of HCV is still obscure and in up to half of patients, the source of HCV infection is not identified (2, 3).

There are contradictory reports in the literature about the role of the intrafamilial transmission (IFT) of HCV. While researchers reported a high serological prevalence of HCV among family members of affected patients, others indicated IFT as a rather infrequent event (2, 4-6). One of the major problems of the earlier studies was the use of the first generations of immunoassays. 

It was demonstrated that body fluids other than blood, including saliva and semen might harbor HCV virus particles (7). Some studies have evaluated HCV genome in the saliva of infected patients by reverse transcription (RT)-PCR and showed that in addition to sexual and vertical transmissions, saliva could be the route of transmission, especially in those of whom no route of infection has been identified (7, 9). Also, transmission of blood or blood related products lead to infection in the majority of cases. While the majority of intravenous drug users become infected by repetitive exposure to shared contaminated injection equipment, its significance in IFT needs to be explored. Other risk factors, including sharing devices and tools, accidental body contact that is related to household living are also taken into consideration by other studies, but the controversy remains. It is relevantly claimed that HCV is less commonly transmitted through some behaviors, including having sex with an infected person, being born to an HCV-infected mother, or sharing of personal tools contaminated with infectious blood (6), but different points of view have been raised by other reports worldwide. Besides, economical/social state rather than education level, has been proposed to affect HCV transmission as considered more recently, but poorly determined. 

Therefore, the present study aimed to evaluate 34 HCV affected families for the rate of transmission, as well as their contributed main risk factors in Fars province, a southern part of Iran. This finding may highlight earlier risk factors in IFT HCV transmission and the propose strategies for controlling the transmission route. 

## Patients and Methods

**The study population and cases**

This cross-sectional study was conducted in Shiraz city, south of Iran. The sampling procedure was performed in a referral medical center of liver diseases in Fars province, Shahid Motahari Liver Clinic affiliated to the Shiraz University of Medical Sciences. The sampling was started in late spring and extended up to the end of the summer, 2013. The study has been performed according to the World Medical Association Declaration of Helsinki and the procedure was approved by the Ethics Committee of the Shiraz University of Medical Sciences, Shiraz, Iran. All participants were requested to fill a written informed consent. All household members of family and close relatives, including parents, brother, sister, brother-in-law and sister-in-law in those families with one HCV index case were invited to participate in the study. They were interviewed thoroughly and demographic data (age, sex, geographical place, education level and socioeconomic state) as well as possible risk factors were gathered. The considered risk factors included: hospitalization, blood transfusion, incarceration, surgery, tools sharing, contact with contaminated stuffs, sex and drug abusing. An arbitrary and conclusive social-economic statement (CSES) based on parents job, income, mean education level and house location was also determined for each family (10, 11). In a clean situation, serum samples were obtained from all subjects.

**Serological and molecular diagnosis of viral spread**

In the first step, to evaluate the exposure rate the presence of total anti-HCV antibodies was determined using fourth generation anti-HCV enzyme linked immunosorbent assay (ELISA) kit (Diapro Inc. Italy) on serum samples according to the manufacturer’s instructions. 

Then, to survey the current molecular state in HCV antibody positive cases, viral RNA genome was extracted from sera by a commercially available kit (Invitek Inc, Germany). For cDNA synthesis, HyperScript^TM^ Reverse Transcriptase (GeneAll Inc, South Korea) was employed while a total of 10 μl of extracted RNA virus was introduced to each reaction tube and random hexamer was added as primer followed by 1 hour incubation at 45^o^C. Then, an in-house Nested-PCR method targeting 5^/^UTR of the genome was employed on sera with positive serologic members, as described before (12). In addition, for more accurate evaluation, a commercial Real-Time PCR detection method, Amplisens HCV-FRT (Russia) was also employed to confirm the presence of viral genome inside the serum (12). Finally, a simple genotyping assay was performed on family members with PCR positive results (in those families with >2 affected members). For this purpose, a gel-based genotype determination kit (AmpliSens HCV-genotype-EPh PCR kit, AmpliSens, Russia) was employed based on recommended instructions.

**Statistical analysis**

The data were presented as mean (±standard deviation) for quantitative data and as frequency or percentage for qualitative data. Two independent sample t test was used to statistically discriminate between HCV positive and negative cases and also between families with one index case with those families with more than 1 positive patient. The Chi-Square test was used to find out the associations between risk factors and status of HCV infection. All data were analyzed with SPSS version 17.0 and P<0.05 was considered as significant difference. 

## Results

In this study, a total of 34 families encompassing 236 members from 9 different cities in Fars province, southern part of Iran were evaluated. Demographic characteristics of 236 enrolled family members, including: age, gender, conclusive socioeconomic status and education levels are presented in Table 1. Total mean ± SEM of age of cases was 35.33 ± 1.03 years and the population sample had a good geographic distribution among cities. 

**Table 1:** Demographic information of our patients (n=236)*

Variables	
Age (mean ± SEM)	
Male	35.42 ± 1.38†
Female	35.22 ± 1.54
Gender	
Male	123 (52.1)‡
Female	113 (47.9)
Conclusive socioeconomic status	
Low	21 (8.9)
Middle	158 (66.9)
High	57 (24.2)
Education status	
Illiterate	17 (7.3)
Elementary education	33 (14.1)
Secondary education	67 (28.6)
High school education	28 (12.0)
Diploma	56 (23.9)
Associate degree	3 (1.3)
Bachelor and higher	30 (12.8)

* We designed a comparative ranked conclusive social-economic status according to job, income, number of peoples in each family, house place and etc. For two patients, the conclusive socioeconomic status was not cleared. For two patients, the conclusive socioeconomic and education status were not cleared.

† mean ± standard deviation;

‡ number (percent)

**Table 2:** Risk factors of our patients (n=236).

**Variables**	**HCV positive N (%)**	**HCV negative N (%)**	**P**
Age (years)*			0.034
<40 (25.30 ± 9.63)	26 (49.1)	118 (65.2)	
≥40 (51.37 ± 8.78)	27 (50.9)	63 (34.8)	
Gender			<0.001
Male	42 (79.2)	81 (44.3)	
Female	11 (20.8)	102 (55.7)	
Non liver diseases			0.058
Yes	13 (24.5)	25 (13.7)	
No	40 (75.5)	158 (86.3)	
Drug abuse			<0.001
Yes	32 (60.4)	9 (4.9)	
No	21 (39.6)	174 (95.1)	
Intravenous drug abuse			<0.001
Yes	21 (39.6)	1 (0.5)	
No	32 (60.4)	182 (99.5)	
Sharing needle			<0.001
Yes	15 (28.3)	2 (1.1)	
No	38 (71.7)	181 (98.9)	
History of incarceration			<0.001
Yes	18 (34.0)	7 (3.8)	
No	35 (3.8)	176 (96.2)	
Blood/derivative transfusion			0.002
Yes	13 (24.5)	16 (8.7)	
No	40 (75.5)	167 (91.3)	
Dental treatment			0.166
Yes	44 (83.0)	135 (73.8)	
No	9 (17.0)	48 (26.2)	
Unprotected sexual activity			0.001
Yes	11 (20.8)	10 (5.5)	
No	42 (79.2)	173 (94.5)	
Tattoo			<0.001
Yes	12 (22.6)	9 (4.9)	
No	41 (77.4)	173 (95.1)	
Acupuncture			0.001
Yes	10 (18.9)	80 (43.7)	
No	43 (81.1)	103 (56.3)	
Sharing food and drink			0.404
Yes	38 (71.7)	120 (65.6)	
No	15 (28.3)	63 (34.4)	
Sharing toothbrush			0.731
Yes	1 (1.9)	5 (2.7)	
No	52 (98.1)	178 (97.3)	
Sharing blade			<0.001
Yes	12 (22.6)	8 (4.4)	
No	41 (77.4)	175 (95.6)	
Sharing other shave devices			<0.001
Yes	20 (37.7)	20 (10.9)	
No	33 (62.3)	163 (89.1)	

BT, blood/derivative transfusion;

* In age category, the mean ±SD of each group is given

Among them, 18 families (52.9%) had at least one new infected member in addition to index case, based on the serologic data for HCV infection (HCV exposure). Of these, 22 new recognized HCV antibody positive cases, 12 members from 11 families also had positive PCR results, which was indicative of active infection as confirmed by 2 different PCR assays.

Overall, comparison of related risk factors in all HCV positive and negative members, according to sociological results, is presented in Table 2. Age, gender, drug abuse, history of incarceration, blood transfusion, sexual activity, shared needles, blood transfusion, tattoo, sharing blades and tools have a significant importance as risk factors. Among all evaluated risk factors, only suffering from non-liver diseases, dental treatment, sharing food/drink or toothbrush and contact with a bloody cloth had no significant difference between HCV positive and negative family members.

**Figure 1:**
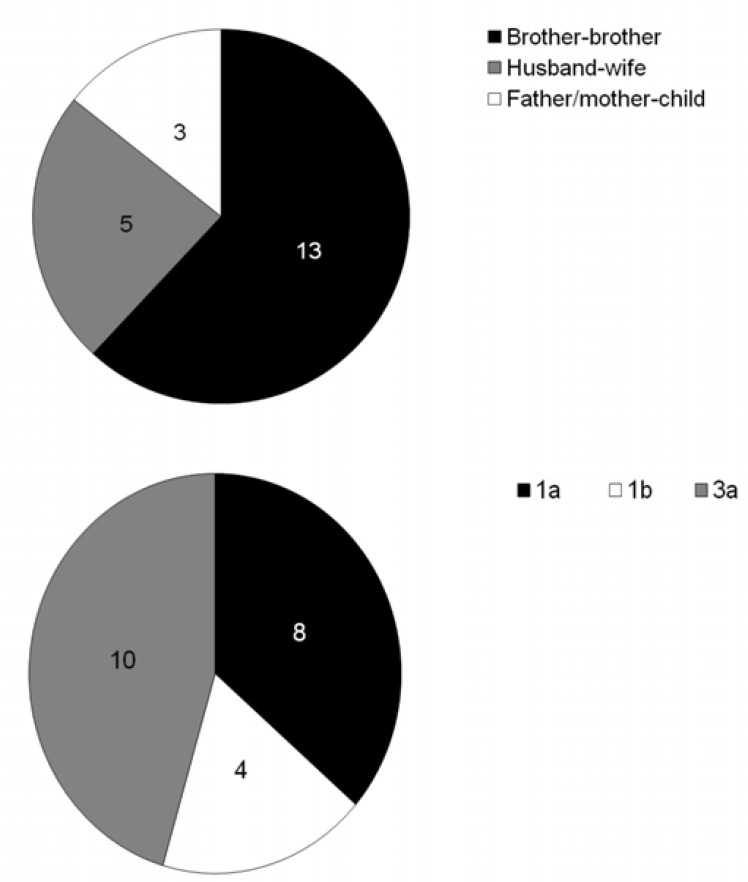
Overall family relationship types (A) and genotype analysis (B) between index cases and affected members in his/her family. The results indicated that 13 affected family exhibited brother-brother transmission route, based on serological study and genotype 3 was the most prevalent virus among infected persons

**Table 3:** The analysis of risk factors for Hepatitis C virus infection among families

**Risk factors**	**HCV** a ** Genome**			**anti-HCV antibody**		
	**A**	**B**	**P**	**A**	**B**	**P**
Non liver diseases	27 (14.9)	11 (20.0)	0.369	19 (14.6)	19 (17.9)	0.491
Drug abuse	23 (12.7)	18 (32.7)	0.001	17 (13.1)	24 (22.6)	0.054
Intravenous drug abuse	10 (5.5)	12 (21.8)	<0.001	6 (4.6)	16 (15.1)	0.006
Alcohol abuse	27 (14.9)	13 (23.6)	0.131	19 (14.6)	21 (19.8)	0.290
History of incarceration	12 (6.6)	13 (23.6)	<0.001	8 (6.1)	17 (16.0)	0.014
Blood Transfusion	20 (11.0)	9 (16.4)	0.293	10 (7.7)	19 (17.9)	0.017
Hospitalization with BT^b^	9 (5.0)	2 (3.6)	0.681	5 (3.8)	6 (5.7)	0.511
Surgery with BT	10 (5.5)	5 (8.1)	0.342	8 (6.1)	7 (6.6)	0.888
Dental treatment	138 (76.2)	41 (74.5)	0.797	98 (75.4)	81 (76.4)	0.854
Unprotected sexual activity	13 (7.2)	8 (14.5)	0.093	7 (5.4)	14 (13.2)	0.036
Tattoo	13 (7.2)	8 (14.5)	0.096	12 (9.2)	9 (8.6)	0.860
Acupuncture	70 (38.7)	20 (35.4)	0.757	49 (37.7)	41 (38.7)	0.877
Sharing food and drink	119 (65.7)	39 (70.9)	0.476	82 (63.1)	76 (71.7)	0.161
Sharing toothbrush	4 (2.2)	2 (3.6)	0.556	4 (3.1)	2 (1.9)	0.563
Sharing needle	9 (5.0)	8 (14.5)	0.016	7 (5.4)	10 (9.4)	0.231
Sharing blade	11 (6.1)	9 (16.4)	0.016	8 (6.1)	12 (11.3)	0.156
Sharing shave devices	28 (15.5)	12 (21.8)	0.272	20 (15.4)	20 (18.9)	0.478

A: families with one positive person; B: families with more than 1 person positive. P: P value.

a Hepatitis C virus,

b Blood Transfusion

**Table 4:** Total and familial effects of conclusive socioeconomic and education states on the number (%) of the Hepatitis C virus transmission, based on virus genome and anti-Hepatitis C virus antibody detection

**Variable**	**Total positive cases**				**Familial** ** comparison**			
	A (PCR)	P	B (serology)	P	C (Unaffected family)	P	D (Affected family)	P
CSES^a^		0.002		0.013		0.298		0.019
Low	10 (22.2)		10 (18.9)		16 (8.8)		6 (5.7)	
Middle	24 (53.3)		30 (56.6)		117 (64.6)		81 (76.4)	
High	11 (24.4)		13 (24.5)		48 (26.5)		19 (17.9)	
Education		0.449		0.646		0.103		0.328
Illiterate	5 (11.1)		5 (9.4)		7 (13)		11 (10.5)	
Elementary education	4 (8.9)		5 (9.4)		5 (9.3)		16 (15.2)	
Secondary education	17 (37.8)		18 (34.0)		20 (37.0)		29 (27.6)	
High school education	4 (8.9)		6 (11.3)		9 (16.7)		13 (12.4)	
Diploma	12 (26.6)		15 (28.3)		7 (13.0)		20 (19.0)	
Associate degree	0 (0)		0 (0)		0 (0)		0 (0)	
Bachelor and higher	3 (6.7)		4 (7.5)		6 (11.1)		15 (14.3)	
Religious degrees	0 (0)		0 (0)		0 (0)		1 (1.0)	

A: Hepatitis C RNA positive; B: anti-Hepatitis C virus antibody positive; C: Family with only one hepatitis C virus genome positive; D, Family with more than one infected person.

a Conclusive socioeconomic state.

Risk factor analysis, among families with one or more than one HCV positive case, according to both serological and RNA evaluations are presented in Table 3. These results demonstrated that based on HCV RNA detection, traditional/intravenous drug abuse (P=0.001), history of jail (P=0.001) and sharing needle or blade (P=0.016) were the significant risk factors indicating differences between families with one or >1 HCV positive members. On the other hand, based on serological detection of total anti-HCV antibody (HCV exposure), intravenous drug abuse (P=0.054), history of jail (P=0.014), blood transfusion (P=0.017) and unprotected sexual activity (P=0.036) were the risk factors that had significant differences between families with one or more than one HCV positive members.

In another part of the study, some degree of correlation between conclusive socioeconomic state (CSES) and intrafamilial transmission of HCV was revealed, as presented in Table 4. In our study, approximately 76% of people were categorized under the medium level of CSES. Our data demonstrated that patients with a middle level of CSES had the highest percentage among PCR and serologic positive members (p=0.002 and p=0.013, respectively). Hence, we found that in families with a middle level of CSES, the spread rate (one new infected member) was significantly higher (p<0.001). In spite of CSES, different education level had no significant correlation with intrafamilial transmission or HCV infection among all members.

In term of the kind of family relationship, possible transmission of virus was higher in brother-brother, spouses, and parent-daughter/son interactions respectively, based on serological findings. For retaliation factor, out of 22 new HCV exposed members, interestingly 13 cases were brothers, while just 5 cases were couples of index cases. However, no possible sisterhood transmission was found in investigated families. The members who had a brother-brother relationship were positive 5.2 and 9.75 times more prone to HCV transmission than members who were spouses (95% CI: 1.3674-19.7741) or parent-daughter/son (95% CI: 2.1613-43.9832). 

At the end, in 11 families that had more than one HCV PCR positive members, genotype was determined in the index case and related new infected member (total 23 persons). The prevalent genotypes were determined 3a (10 cases), 1a (8) and 1b (4) among infected members respectively. 

## Discussion

Among the proposed non-clarified mode of HCV transmission, interfamilial transmission (IFT) suggest to have a special place and further investigation is demanded. As a controversial issue, the exact role of close household interactions, sexual contact between spouses, sharing tools and type of relativeness as risk factors were remained to be clearly elucidated (13). More information in this era will present new ideas for a better control of HCV transmission. 

In the present study, conducted in the south of Iran, the rate of IFT and well-defined risk factors in addition to the impact of CSES status and educational level were investigated among 34 families with one HCV index case. 

The rate of HCV transmission among family members is controversial and household contact risk factors remain to be delineated more, even in spouses (14). In similar studies from our country, it was demonstrated that IFT plays no significant role in HCV spread nor sexual contact (14). Some previous studies relied on determining the HCV infection based on just serologic assays, which seems ambiguous for the epidemiological study (15). Such reports, although informative for epidemiologic study, indicate that a conclusion based on a molecular testing is crucial (16). 

The evaluation of HCV genome in relatives by Forbi, et al. demonstrated that intrafamilial transmission is a common route of HCV spread in the communities (17). In contrast, recently Lu, et al. in China evaluated 1,050 subjects in a high prevalence area for both hepatitis B virus (HBV) and HCV infections. They reported that in spite of the role of IFT for HBV spread, HCV transmission is commonly associated with the use of contaminated medical equipment (18). Also, Ndong-Atome, et al. showed that in central Africa the HCV transmission is possibly iatrogenic rather than intrafamilial or sexual (19). In earlier studies in our area, the significant role of IFT in HCV spread was refused, though challenging questions still need to be solved (14, 15, 20). In our study, the high rate of HCV exposure (18 out of 34 families) was recognized based on serological state. Furthermore, 11 out of 18 families revealed to harbor active viral infection as documented by PCR. That was simply indicative of the importance of IFT for hepatitis C virus spread in our era.

Among the considered risk factors, interspousal and sexual transmission of HCV showed less significance in spite of common imagination. Although sexual transmission of HCV suggests a greater potential of male-to-female transmission (6), other risky behaviors such as sharing needle, blade, shaving device and tooth brush are proposed to be a significant route of HCV transmission, as demonstrated in our study. While five possible interspousal transmission events occurred, our findings demonstrated that it has no similar importance as brother-brother transmission. These results are in line with studies that implied extremely low risk of sexually acquiring HCV infection (21, 22). The rare interspousal transmission of HCV among families was also indicated before by Boonyarad, et al. (23). Ranjbar, et al. also showed that interfamilial transmission of HCV is a less common event compared to the HBV infection (24). From our data, it seems that brother-brother transmission is more common in the southern part of Iran. This may be due to some habits like sharing personal tools and partnership in outside risky behaviors. This kind of report has not been presented before by others. 

As a social-familial factor, CSES was somehow shown to be a significant factor in HCV infection and even in IFT spread. There is disagreement between researchers about the relationship between CSES status and viral transmission. Akbar, et al. reported that in contrast to HCV infection, low socioeconomic status was a strong risk factor for HBsAg seropositivity (25). In a study by Awadalla, et al., significant correlation was found between HCV seropositivity and lower socio-demographic state of the blood donors in Egypt (10). This correlation was profoundly demonstrated elsewhere by others (26). 

The prevalence of HCV genotypes among studied families mimicked country pattern, as genotype 3a and 1a were more prevalent among the population. 

Although our findings provide some informative results about IFT transmission of HCV, a major limitation should be noticed before the final interpretation. The lack of sequence analysis and phylogenetic survey make the findings less reliable to determine the real transmission of HCV among members. Moreover, PCR method was just performed on ELISA positive cases, so the recent acute HCV infection is assumed to be missing from the study. 

In conclusion, HCV intrafamilial transmission was common in the population and among relatives, brother to brother transmission was the most prevalent way. Furthermore, having a history of intravenous drug abuse, incarceration, personal tools sharing and sex were significant risk factors. Also, the CSES may be a determinant factor in the epidemiology of HCV.
